# Elucidating the Impact of Li_3_InCl_6_-Coated LiNi_0.8_Co_0.15_Al_0.05_O_2_ on the Electro-Chemo-Mechanics of Li_6_PS_5_Cl-Based Solid-State Batteries

**DOI:** 10.1021/acs.chemmater.4c00515

**Published:** 2024-06-13

**Authors:** Feng Jin, Laras Fadillah, Hung Quoc Nguyen, Torgeir Matre Sandvik, Yu Liu, Adrián García-Martín, Elena Salagre, Enrique G. Michel, Dragos Stoian, Kenneth Marshall, Wouter Van Beek, Günther Redhammer, Mir Mehraj Ud Din, Daniel Rettenwander

**Affiliations:** † Department of Material Science and Engineering, NTNU Norwegian University of Science and Technology, Trondheim 7491, Norway; ‡ Departamento de Fisica de la Materia Condensada, Facultad de Ciencias, Universidad Autónoma de Madrid, Madrid 28049, Spain; § Condensed Matter Physics Center (IFIMAC), Universidad Autónoma de Madrid, Madrid 28049, Spain; ∥ Swiss-Norwegian Beam Lines at European Synchrotron Radiation Facility, 71 Avenue des Martyrs, Grenoble 38000, France; ⊥ Department of Chemistry and Physics of Materials, University of Salzburg, Salzburg 5020, Austria; # Christian Doppler Laboratory for Solid-State Batteries, NTNU Norwegian University of Science and Technology, Trondheim 7491, Norway

## Abstract

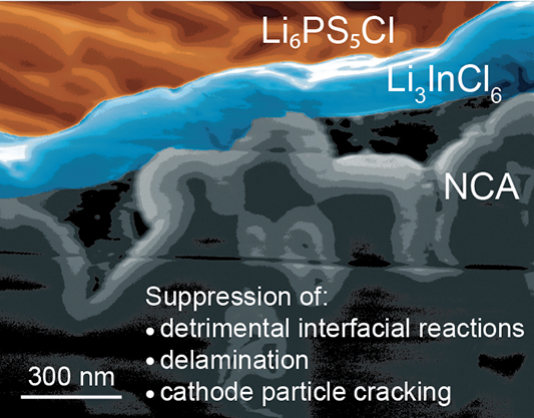

Li_6_PS_5_Cl has attracted significant attention due to its high Li-ion conductivity and processability, facilitating large-scale solid-state battery applications. However, when paired with high-voltage cathodes, it experiences adverse side reactions. Li_3_InCl_6_ (LIC), known for its higher stability at high voltages and moderate Li-ion conductivity, is considered a catholyte to address the limitations of Li_6_PS_5_Cl. To extend the stability of Li_6_PS_5_Cl toward LiNi_0.8_Co_0.15_Al_0.05_O_2_ (NCA), we applied nanocrystalline LIC as a 180 nm-thick protective coating in a core–shell-like fashion (LIC@NCA) via mechanofusion. Solid-state batteries with LIC@NCA allow an initial discharge specific capacity of 148 mA h/g at 0.1C and 80% capacity retention for 200 cycles at 0.2C with a cutoff voltage of 4.2 V (vs Li/Li^+^), while cells without LIC coating suffers from low initial discharge capacity and poor retention. Using a wide spectrum of advanced characterization techniques, such as operando XRD, XPS, FIB-SEM, and TOF-SIMS, we reveal that the superior performance of solid-state batteries employing LIC@NCA is related to the suppression of detrimental interfacial reactions of NCA with Li_6_PS_5_Cl, delamination, and particle cracking compared to uncoated NCA.

## 1 Introduction

Solid-state batteries are considered as one of the most promising next generations of energy storage technologies with a potential to revolutionize electric vehicles (EVs), due to their high energy density and intrinsic safety.^1^ Here, the solid electrolyte is key since it allows the utilization of Li or Si as anode and the possibility to have bipolar cell configurations. Hence, several solid electrolytes have been explored, whereby most of them fall into two groups:^2^ (i) oxide-based solid electrolytes, such as garnet-type Li_7_La_3_Zr_2_O_12_ and NaSICON-type Li_1+*x*_Al_*x*_Ti_2-*x*_(PO_4_)_3_ and (ii) sulfides, such as thio-LISICON-type Li_10_GeP_2_S_12_ (LGPS) and argyrodite-type Li_6_PS_5_Cl (LPSCl). Of most solid electrolytes, LPSCl is considered one of the promising candidates, owing to its high ionic conductivity, favorable mechanical performance, and ease in processing.^3^ However, there are significant challenges associated with the energy density, rate capability, and capacity retention of solid-state batteries employing LPSCl.^4^ These challenges arise from the interfacial contact-loss during electrochemical cycling as a consequence of volumetric changes in electrodes and the electrochemical stability limitations in the preferred operation voltage range causing undesirable side reactions with high-voltage Ni-rich cathodes, giving rise to fast capacity fade, thus impeding their ultimate commercial realization.^5^

To mitigate the challenges associated with interfacial degradation caused by detrimental secondary reactions, the application of a protective coating on the surface of cathode particles is regarded as a productive mitigation strategy.^6^ A considerable amount of research has been performed to explore suitable coating materials, such as oxides, e.g., ZrO_2_,^7^ HfO_2_,^8^ Li_2_ZrO_3_,^9^ and LiNbO_3_,^10^ and phosphates, e.g., Li_3_PO_4_^11^ and LiZr_2_(PO_4_)_3_.^12^ High-voltage cathodes coated with metal oxides and phosphates show improved specific capacity and capacity retention compared with uncoated counterparts in thiophosphate-based SSBs. However, these coating additives exhibit either limited Li-ion conductivity (<10^–4^ S/cm), or insufficient stability against high-voltage cathode.

Halides, in particular, chlorides, such as Li_3_InCl_6_ (LIC),^13^ Li_3_YCl_6_,^14^ Li_3_TiCl_6_,^15^ and Li_3–*x*_M_1–*x*_Zr_*x*_Cl_6_ (M = Y, Er),^16^ have gained considerable attention by combining both high Li-ion conductivities (>0.1 mS/cm) and high-voltage stability (>4.2 V vs Li/Li^+^). Nevertheless, their instability when paired with a Li anode^17^ and relatively high production costs limit their application as bulk solid electrolytes. However, when used as a catholyte or as a specific coating additive for cathodes, the production costs and instability of halide-based solid electrolytes against Li are less significant, making them a promising alternative. To avoid direct contact between sulfide electrolytes and high-voltage cathodes, Yu et al.^18^ proposed a dual electrolyte approach, where LPSCl is used as the separator and LIC as the catholyte. However, the assembled Li–In|LPSCl|NMC cell has been only functional at elevated temperatures (60 °C), due to the large amount and higher thickness of LIC that result in higher cell impedance. To overcome kinetic limitations Ye et al. used LPSCl and LIC as the catholyte in the NMC composite cathode by using a slurry coating method.^19^ They observed an improved initial discharge capacity in their Li–In|LPSCl|NMC cell when LIC is introduced. However, the long-term electrochemical performance appears to be relatively poor (77.4% capacity retention after 100 cycles) even at a lower C-rate of 0.1C. The poor cycling performance is likely due to the insufficient covering of NMC particles by LIC during slurry processing causing an irreversible capacity loss as a consequence of electrolyte decomposition. Contrary to these reports, recent studies questioned the compatibility of LIC and LPSCl in Ni-rich-based cathode cells, in general. For example, Koç et al. found that at a cell potential ≥4.23 V (vs Li/Li^+^), the decomposition of LIC takes place, as well as a quadruple increase in the interfacial resistance between LIC with LPSCl in a bilayer configuration at 80 °C, indicating not only electrochemical but also chemical instability.^20^ This chemical incompatibility between LIC with LPSCl has been proposed to translate into the formation of a passivation interlayer, which leads to degraded electrochemical performance of up to 50% upon cycling.^21^ This chemical instability has been throughout studied subsequently by Rosenbach et al.^20^ using FIB-SEM combined with TOF-SIMS. They confirmed that LIC with LPSCl is chemically unstable but also highlighted that this degradation is predominant when NMC, LPSCl, and LIC forms triple junction, due to the potential catalytic nature of the cathode. However, they considered their interpretation of the findings as highly speculative. Hence, the stability of LIC with LPSCl is needed to be further elaborated. Despite this controversial discussion, we conclude that LIC can be used as coating material if (i) triple junction between LIC, LPSCl, and Ni-rich cathodes is avoided and (ii) the cutoff voltage is chosen below 4.2 V (vs Li/Li^+^).

**Figure 1:**
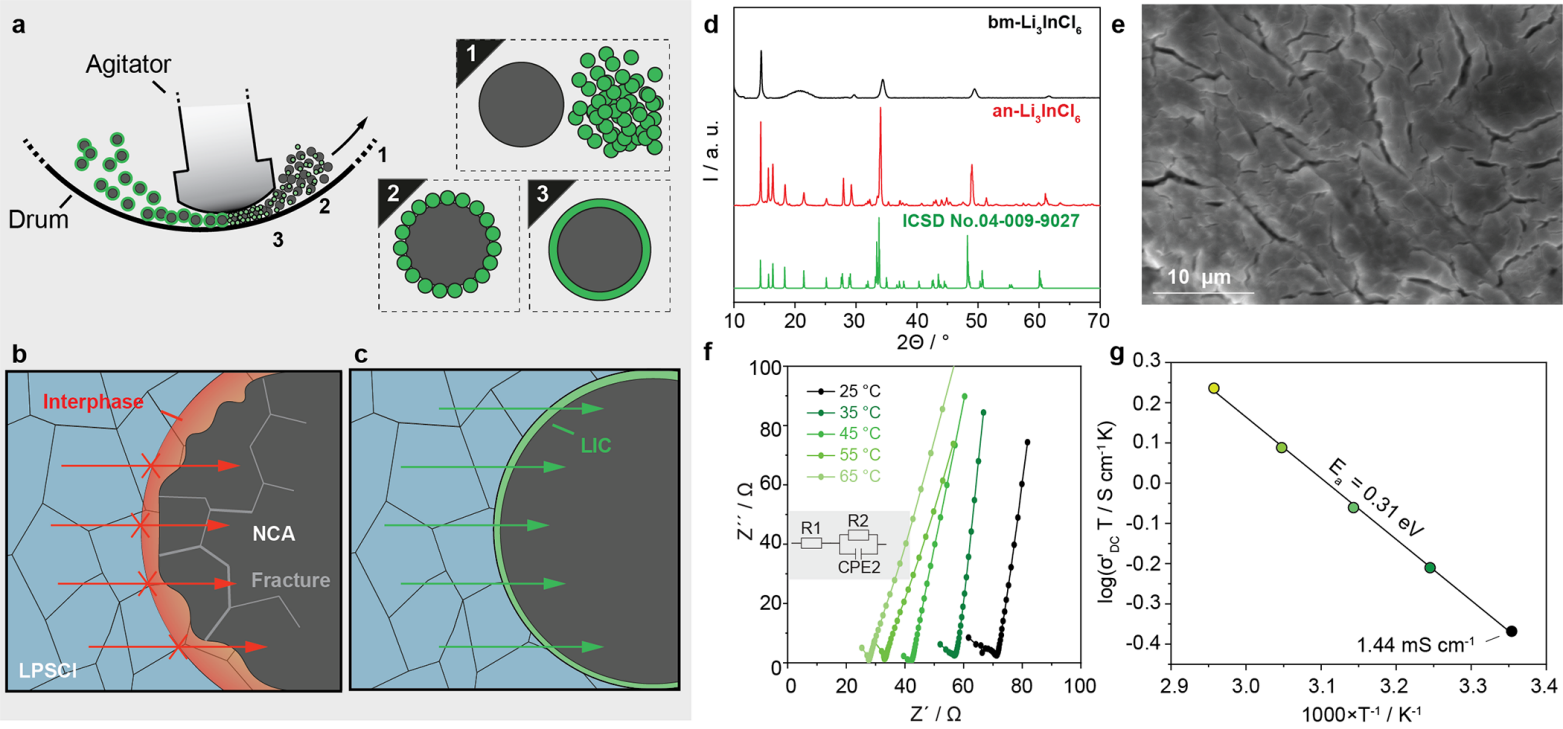
(a) Schematic illustration of mechanofusion and its working principles. (b) Schematic illustration of pristine NCA | LPSCl showing a chemical degradation and formation of resistive CEI at the interface due to parasitic reactions causing hindrance to Li-ion transport. (c) Schematic illustration of LIC@NCA | LPSCl interface representing a stable Li-ion transport without any electrochemical degradation at the interface and the more roundish surface of cathode particles due to the mechanofusion process. (d) XRD patterns of ball milled and annealed LIC powders. (e) SEM images of an-LIC. (f) Nyquist plots of an-LIC in a frequency range from 7 MHz to 1 Hz under various temperatures with the equivalent circuit used to fit spectra presented in the inset and (g) its corresponding Arrhenius plot showing a linear increase in Li-ion conductivity with temperature with an *E*_a_ of 0.31 eV.

Herein, we report on a core–shell-structured cathode composite (Figure 1a), where LiNi_0.8_Co_0.15_Al_0.05_O__2__ (NCA) particles (core) are coated by a 180 nm-thick pinhole free LIC layer (shell), using a scalable mechanofusion process. Due to the LIC coating interfacial decomposition reactions of LPSCl, interfacial delamination and particle cracking, as observed for uncoated NCA-based SSBs, are suppressed (Figure 1b,c), thus facilitating a specific capacity enhancement of about 80 mAh/g for C-rates (0.1C, 0.2C, 0.5C, and 1C) and improved long-term cell cycling.

## 2 Results and Discussion

### 2.1 Development of Nanoscale LIC as Coating Material by High-Energy Ball Milling

LIC was synthesized based on a convenient two-step procedure, comprising mechanochemical mixing via high-energy ball milling and subsequent annealing. The corresponding XRD patterns for LIC before and after annealing are shown in Figure 1d. Both patterns can be indexed with monoclinic rock-salt structure (space group: *C2*/*m*). No phases other than LIC can be observed. The surface morphology as shown in the SEM image in Figure 1e reveals the formation of elongated particles of LIC. After annealing, LIC shows narrow reflections indicating an increased crystallinity, around 82.5%. To determine the Li-ion conductivity of LIC, impedance spectroscopy measurements in a temperature range between 25 and 65 °C were performed. The corresponding Nyquist plots are shown in Figure 1f, revealing one electrical relaxation process: an onset of a high-frequency semicircle and a spike toward low frequencies. Due to the low amount of high-frequency data points, the resistive part of the arc has been simply fitted by a resistance element, which can be attributed to the total Li-ion conductivity of the LIC pellet. The low-frequency spike is typically related to the electrodes. Adding an R|CPE element improves the fit due to the nonideality of the blocking electrodes. The total Li-ion conductivity of LIC at room temperature was calculated to be 1.44 mS/cm, which is similar to the values reported in the literature.^13^ In Figure 1g the temperature dependence of the Li-ion conductivity is shown. It follows Arrhenius behavior according to σ*T* = σ_0_ exp(−*E*_a_/(*k*_B_*T*)), where σ_0_ represents the pre-exponential factor. The activation energy *E*_a_ is 0.31 eV, which is similar to values reported previously.^13^

### 2.2 Applying LIC as a Coating on NCA Particles via Mechanofusion

**Figure 2:**
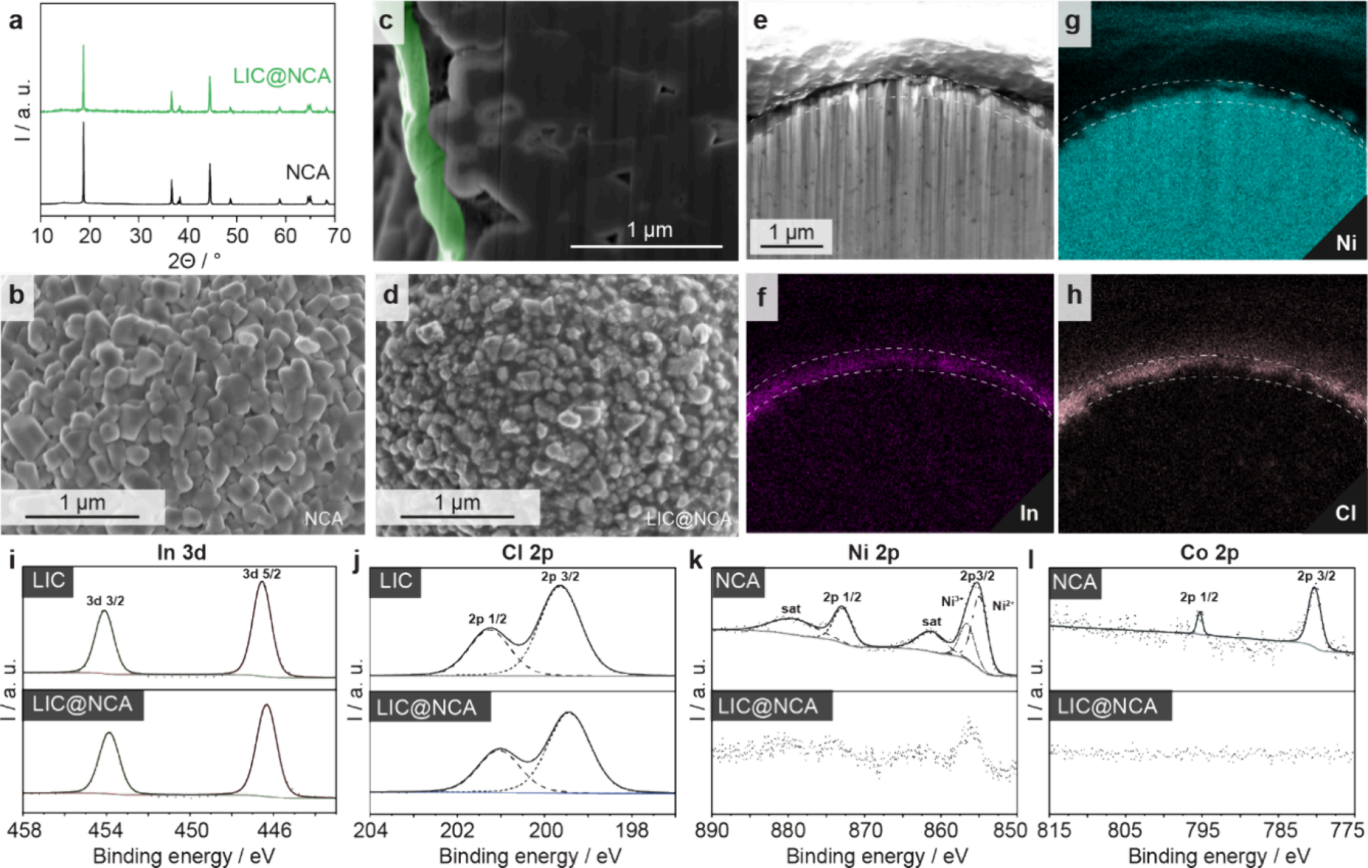
(a) XRD pattern collected from the pristine NCA and LIC@NCA particles. SEM images of pristine NCA (b) and LIC@NCA particles (d). (c) Cross-sectional plasma focused ion beam-scanning electron microscope facility (PFIB-SEM) image of LIC@NCA showing LIC-coated NCA surface. (e) Cross-sectional FIB-SEM image of LIC@NCA and corresponding EDS mapping (f–h). In 3d (i) and Cl 2p (j) XPS spectra for LIC and LIC@NCA and Ni 2p (k) and Co 2p (l) pristine NCA and LIC@NCA.

LIC was coated around NCA particles via a mechanofusion technique (LIC@NCA). Potential changes in phases and crystallinity were further studied via XRD. In Figure 2a, the XRD pattern of LIC@NCA in comparison with NCA is shown. After the coating process, the LIC@NCA was found to retain the pristine layered structure with space group *R*3*m*. No reflections from the LIC were apparent, which may originate from the low weight fraction of the LIC (see Figures S2–S4 and Tables S1–S4 for details). Compared to the pristine NCA, LIC@NCA showed a subtle decrease in cell volume from 101.03 to 100.97 Å^3^. Furthermore, the degree of Ni^2+^/ Li^+^ cation mixing (based on the *c*/*a*) remained similar for both samples, indicating that the crystal structure of NCA remains unaltered after coating with LIC. To prove the successful surface coating, SEM studies, on both pristine NCA and LIC@NCA, were conducted. Figure 2b and Figure S5a depict the magnified SEM images of pristine NCA particles, where secondary NCA particles can be clearly observed. In contrast, the magnified SEM images (Figure 2c and Figure S5b) of LIC@NCA show an altered surface morphology with LIC nanoparticles covering the NCA particle surface suggesting a successful coating. The high shear forces created inside the mechanofusion container by high rotational speeds not only cause the particle rounding and coating of LIC nanoparticles onto the NCA particles surface but also enable homogeneous and densified LIC coating. To evaluate the uniformity and density of the LIC coating, cross-sectional SEM studies have been performed. As shown in Figure 2d, a thick, dense and uniform LIC coating of thickness around 180 nm is evident. Further evidence is given by EDS mapping showing a uniform Cl and In distribution around the NCA particles (Figure 2e). Moreover, the size distribution of mechanofused LIC@NCA particles remains similar to that of pristine NCA, indicating that the cathode particles retained their original size without being milled or experiencing fracture during intense mixing process (Figure S6).

The surface chemistry of LIC, pristine NCA and LIC@NCA was studied via XPS as shown in Figure 2. The In 3d spectrum displays a doublet with a single component and peaks located at 453.8 (In 3d_3/2_) and 446.2 eV (In 3d_5/2_) (Figure 2i). The Cl 2p spectrum also displays a doublet with a single component with peaks at 201.2 (Cl 2p_1/2_) and 199.5 eV (Cl 2p_1/2_) (Figure 2j). These components can be assigned to the In^3+^ and Cl^–^ chemical bonding states in LIC, respectively. Ni 2p and Co 2p core levels from the bare NCA cathode were studied, as well. According to the spectrum of Ni 2p (Figure 2k), two components appear at 854.6 and 856.6 eV, assigned to Ni^2+^ and Ni^3+^, respectively,^22^ an indication of mixed valence.^22,23^ The observation of Ni 2p and Co 2p core levels from LIC@NCA is difficult because the surface sensitivity of XPS significantly decreases their intensity. In turn, the observation of these peaks in LIC@NCA (Figure 2i,j) with a similar line shape (in the case of Ni 2p) and with a strongly damped intensity in both cases, compared with pristine NCA (Figure 2k,l), indicates that NCA is shielded by LIC, which gives further evidence of the successful coating.

### 2.3 Electrochemical Evaluation

To evaluate the functionality of LIC as a protective coating the electrochemical performance in solid-state batteries using NCA (SSB-NCA) and LIC@NCA (SSB-LIC@NCA) has been tested (see details in the Experimental Section and Figure S7).

**Figure 3:**
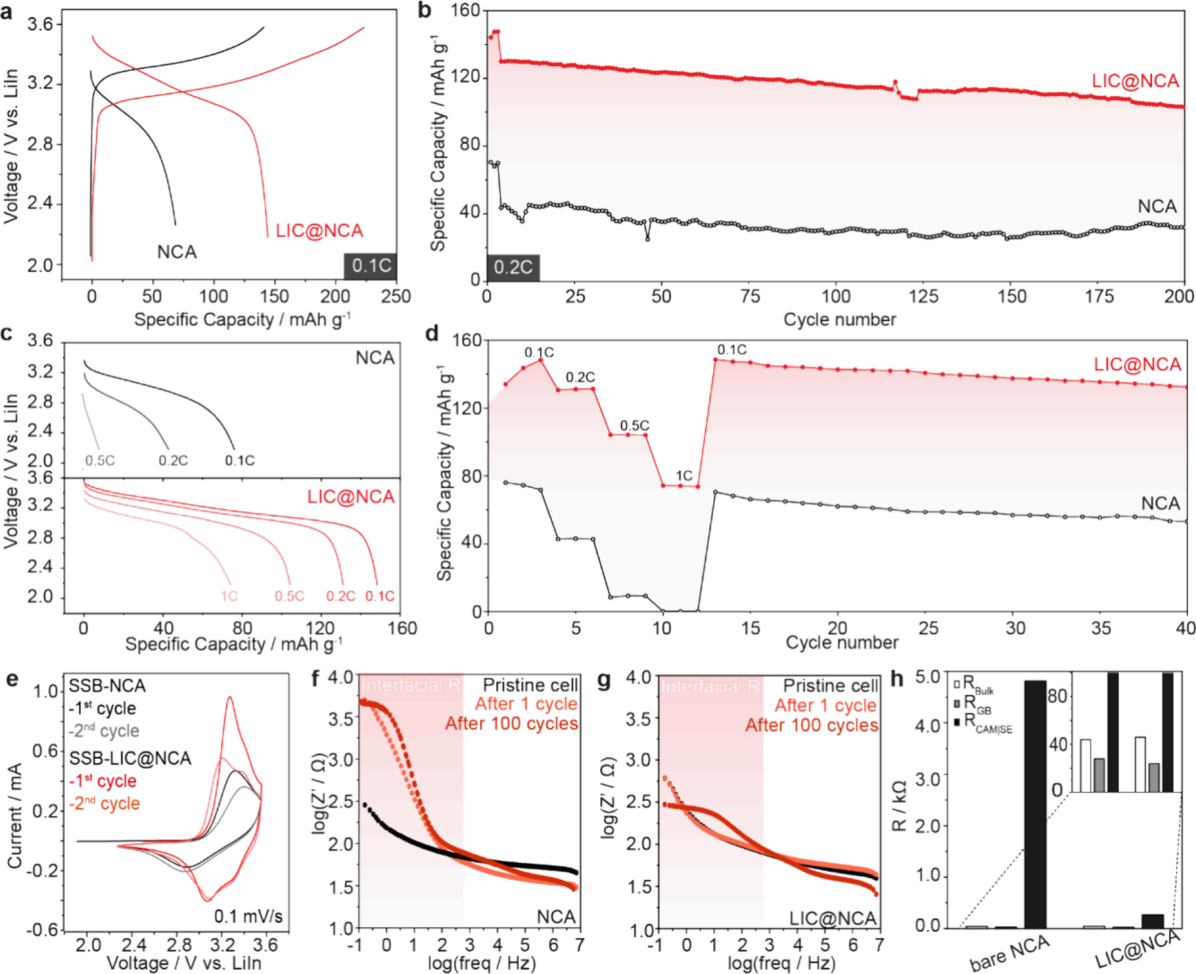
(a) Voltage profiles of the pristine NCA and LIC@NCA for the first cycle at 0.1C and (b) corresponding long-term cycling behavior at 0.2 C. (c, d) Rate capabilities and corresponding voltage profiles from 0.1 to 1.0 C. (e) CV curves of the first two cycles for SSB-NCA and SSB-LIC@NCA cells with a scan rate of 0.1 mV/s. Bode plots of the electrochemical impedance of SSB cells based on cathodes with pristine NCA and LIC@NCA (f) before cycling and (g) after cycling (1 cycle and 100 cycles). (h) Summary of the impedance results of cells after 100 cycles. All tests have been performed at 25 °C.

The initial charge and discharge curves of SSBs recorded at 0.1C (1C = 160 mAh/g) with a cutoff voltage of 4.2 V (vs Li/Li^+^) are presented in Figure 3a. It is evident that for SSB-LIC@NCA, the polarization during charge and discharge is effectively suppressed and the discharge capacity is significantly increased (from 71.8 mAh/g for SSB-NCA to 148.3 mAh/g for SSB-LIC@NCA). Moreover, SSB-LIC@NCA shows significantly more stable long-term cycling at around 133.6 mAh/g and 80.1% capacity retention at 0.2 C compared with SSB-NCA with around 40 mAh/g for 200 cycles (Figure 3a–d). The obtained initial discharge capacity of SSB-LIC@NCA is similar to cells tested based on commercial LiNbO_3_-coated NCA (purchased from NEI) (see Figure S9) as well as to what has been reported previously for cells operated up to a cutoff voltage of 4.2 V (vs Li/Li^+^).^24−26^

In contrast to our expectations, a further increase in the cutoff voltage to 4.4 V (vs Li/Li^+^) allows for discharge capacities as high as 174 mAh/g at 0.1C and 84% capacity retention at 0.2C for 50 cycles (Figure S10). This points toward extended electrochemical stability of LIC exceeding reported computationally predicted values.^17^ The origin of this extended stability (see XPS data in Figure S11 and SI note II) will be, however, subject of subsequent studies. The charge/discharge profiles together with the cycle performance under different C-rates (i.e., 0.1, 0.2, 0.5, and 1C) are shown in Figure 3b,c, respectively. It is evident that SSB-LIC@NCA exhibits a higher capacity at any current density (about 80 mAh/g at any rate tested). In detail, the initial charge capacity for LIC@NCA compared to NCA is markedly increased from 71.8 to 148.3 mAh/g at 0.1 C, from 42.8 to 133.6 mAh/g at 0.2 C, from 8.4 to 104.2 mAh/g at 0.5C, and from 0.2 to 74.3 mAh/g at 1 C.

To better understand redox behaviors during charge and discharge, cyclic voltammetry (CV) has been performed The corresponding CV profiles of SSB-NCA (black and gray lines) and SSB-LIC@NCA cells recorded at a scan rate of 0.1 mV s^–1^ for the first two cycles are shown in Figure 3e. For the SSB-NCA cell, there is only one peak emerging at around 3.35 V during positive scan in the first cycle. In the second cycle, the peak shifts to higher voltage at around 3.46 V. The redox reaction gap (Δ*V*) between the oxidation peak and the reduction peak of SSB-NCA in the first cycle is 0.580 V and increases in the second cycle to 0.583 V. The large Δ*V* is the indicative of higher cell polarization, which can be related to a higher interface resistance. In contrast, all the characteristic peaks related to the phase transitions for NCA during lithiation and delithiation, i.e., hexagonal (H1) to monoclinic (M), monoclinic (M) to hexagonal (H2), and hexagonal (H2) to hexagonal (H3),^27^ are observed in SSB-LIC@NCA cells. Due to the irreversible phase change during the first reaction, the oxidation peak of the second cycle is slightly different from the oxidation peak of the first cycle. The Δ*V* between the oxidation peaks and the reduction peaks of SSB-LIC@NCA in the first cycle is 0.2 V, and in the following cycle, the Δ*V* is gradually reduced to 0.13 V indicating that the interface resistance in SSB-LIC@NCA is relatively small. The absence of the characteristic peaks in SSB-NCA could be related to the higher internal cell resistance, which leads to an overpotential gradient across the composite cathode and hence to the formation of a local SOC variation, resulting in the merging of peaks in the CV spectra.^28^

The larger polarization of SSB-NCA compared to SSB-LIC@NCA (Figure 3a) could be related to the instability of LPSCl when in direct contact with NCA, which leads to its decomposition and formation of highly resistive interphases.^25^ The formation of such resistive interphases is evident from the impedance spectroscopy analysis conducted for both pristine NCA and LIC@NCA before and after cycling. The corresponding impedance spectra of the cells in the bode plot representation are shown in Figure 3f,g. Both cells exhibit two characteristic plateaus, whereby the high-frequency plateau represents the resistance of the electrolyte and the low-frequency plateau the interfacial impedance between the electrolyte and the electrode material. Here, the bulk resistances for SSB-NCA and SSB-LIC@NCA are similar (∼44 Ω), while the interface resistance for SSB-LIC@NCA increased slightly (∼20 Ω), which is associated with the LIC coating (Figure 3h). The total resistance of the cell is about 120 Ω, suggesting that the overpotential of the cell is dominated by the cell resistance. Despite the higher initial cell resistance of SSB-LIC@NCA, the interfacial resistance of SSB-NCA significantly increased after the first cycle, which can be associated with interfacial degradation processes. This increase is even more pronounced after 100 cycles, where the resistance reaches values around 4813 Ω in contrast to SSB-LIC@NCA with a resistance of only 267 Ω (see Figure 3h). An incremental increase in the interfacial resistance of SSB-LIC@NCA suggests that LIC coating can hinder or at least slow down interfacial degradation processes and consequently improves the specific capacity and rate performance.

### 2.4 Origin of Cell Performance Improvement

To understand the interfacial degradation processes and the mechanism of suppression promoted by the LIC coating, the morphological and chemical degradation processes have been studied via operando synchrotron XRD, SEM and PFIB-SEM, and XPS.

**Figure 4:**
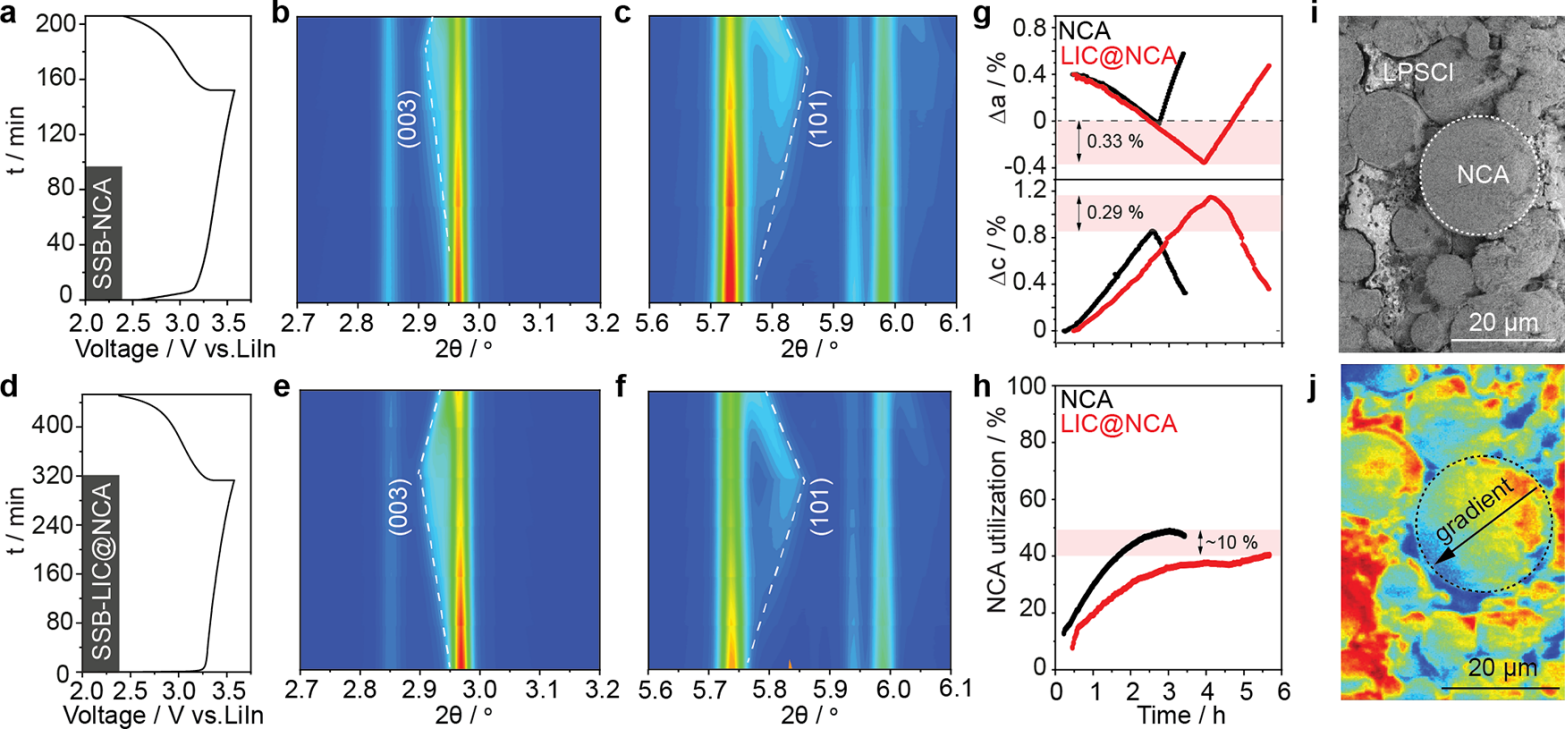
Operando synchrotron XRD data was collected from SSB-NCA (a–c) and SSB-LIC@NCA (d–f) cells in the initial cycle. Contour plots show the evolution of Bragg reflections and the corresponding voltage profile as a function of *x*(Li). Relative changes of lattice parameters of SSB-NCA and SSB-LIC@NCA during the initial cycle. Note that values are the statistically averaged results of many particles inside the operando cells. Relative changes of lattice parameters during the initial cycle (g). Active material utilization during first cycle in SSB-NCA and SSB-LIC@NCA (h). Cross-sectional PFIB-SEM image (i) of charged LIC@NCA cathode composite and its corresponding TOF-SIMS image based on Li^+^(j).

Operando synchrotron XRD data recorded during the first cycle show similar behavior in the structural and phase evolution of NCA for SSB-NCA and SSB-LIC@NCA (Figure 4a–f; see details about the analysis in Supporting Information Note 1 and Figures S12–S15). In both cells, SSB-NCA and SSB-LIC@NCA, distinct shifts of the (003) and (101) NCA reflections can be observed during operation. During charge, the *a*-axis lattice parameter decreases, while the *c*-axis lattice parameter increases (Figure 4g) and vice versa during discharge (details in Figure S10). Interestingly, lattice parameter changes are more prominent for SSB-LIC@NCA (NCA: *a* = 0.422%, *c* = 0.86%; LIC@NCA: *a* = 0.755%, *c* = 1.15%), indicating that more Li has been extracted from NCA during charge; hence, higher specific capacities should have been achieved; this is however not the case. By taking a closer look at the diffraction pattern, it is evident that a major part of NCA did not undergo structural changes. To quantify the NCA utilization in composite cathodes, Rietveld analysis was performed. The refinements revealed that only 50 and 40% of NCA has been utilized in SSB-NCA and SSB-LIC@NCA, respectively (Figure 4h). This partial utilization of NCA could be associated with low tortuosity and interface contact challenges in the composite cathode.^29,30^ The heterogeneous state-of-charge distribution within the composite cathode, but even within single particles, is also evident from TOF-SIMS analysis performed on the composite cathode cross section of SSB-LIC@NCA (Figure 4i,j). Interestingly, the NCA utilization is lower for LIC@NCA. We hypothesize that the lower NCA utilization in SSB-LIC@NCA is related to the additional barrier for electron transport introduced by the LIC coating. Despite the lower utilization of NCA in SSB-LIC@NCA, its overall performance is superior compared to SSB-NCA (see Figure 3), which is related to the mitigation of parasitic side reactions. These side reactions result in a highly resistive interphase that increases cell polarization and eventually leads to a cutoff voltage at a lower state-of-charge explaining the lesser changes in cell parameters.^31^

**Figure 5:**
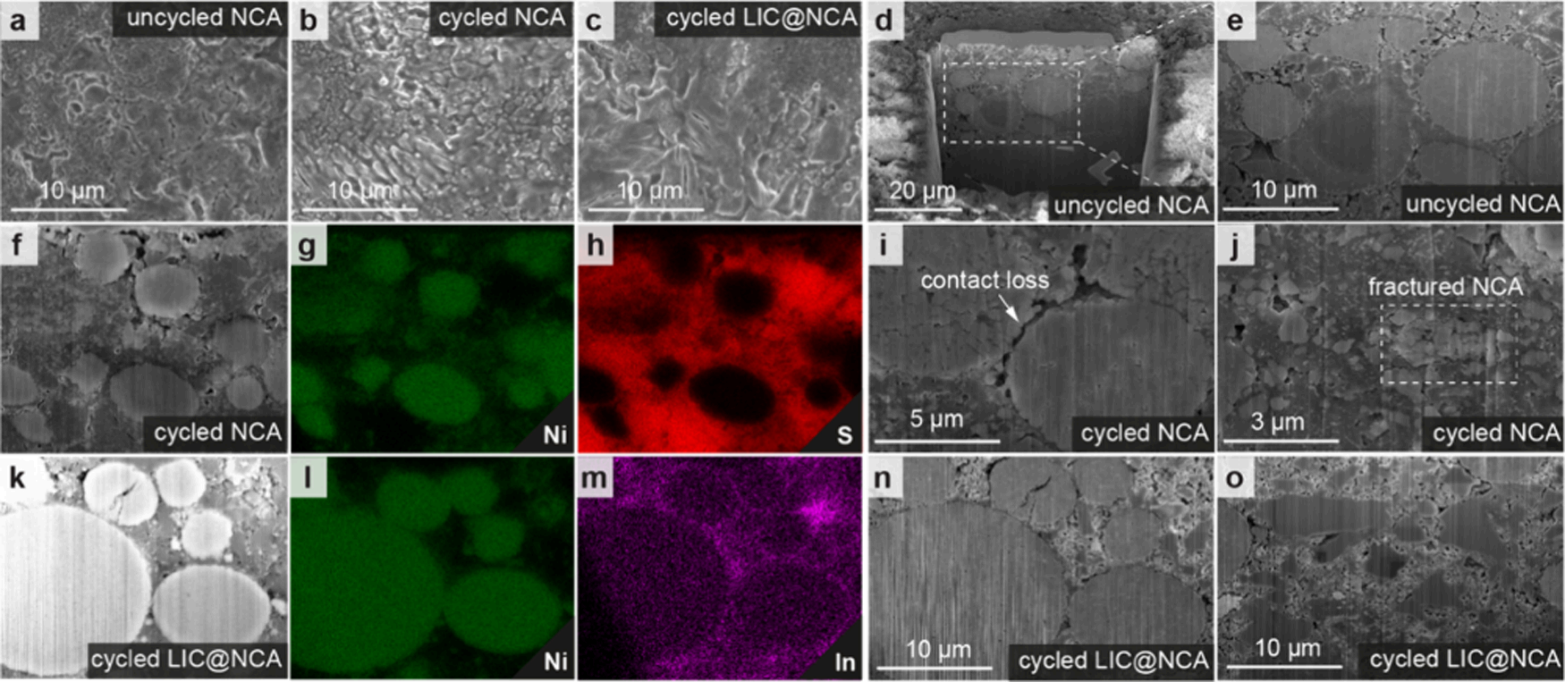
Top view (a–c) SEM images of cathodes with pristine NCA and LIC@NCA before and after cycling (200 cycles). PFIB-SEM images of uncycled NCA and LPSCl composite (d, e). PFIB-SEM images of cycled NCA and its corresponding EDS mapping (f–h). Cross-sectional PFIB-SEM images of cycled NCA showing contact loss and NCA particles fracturing (i, j). PFIB-SEM image of cycled LIC@NCA and its corresponding EDS mapping (k–m) confirming the LIC coating on NCA particles. Cross-sectional PFIB-SEM images of cycled LIC@NCA showing intimate particle contacts and no fracturing of NCA particles (n, o).

**Figure 6:**
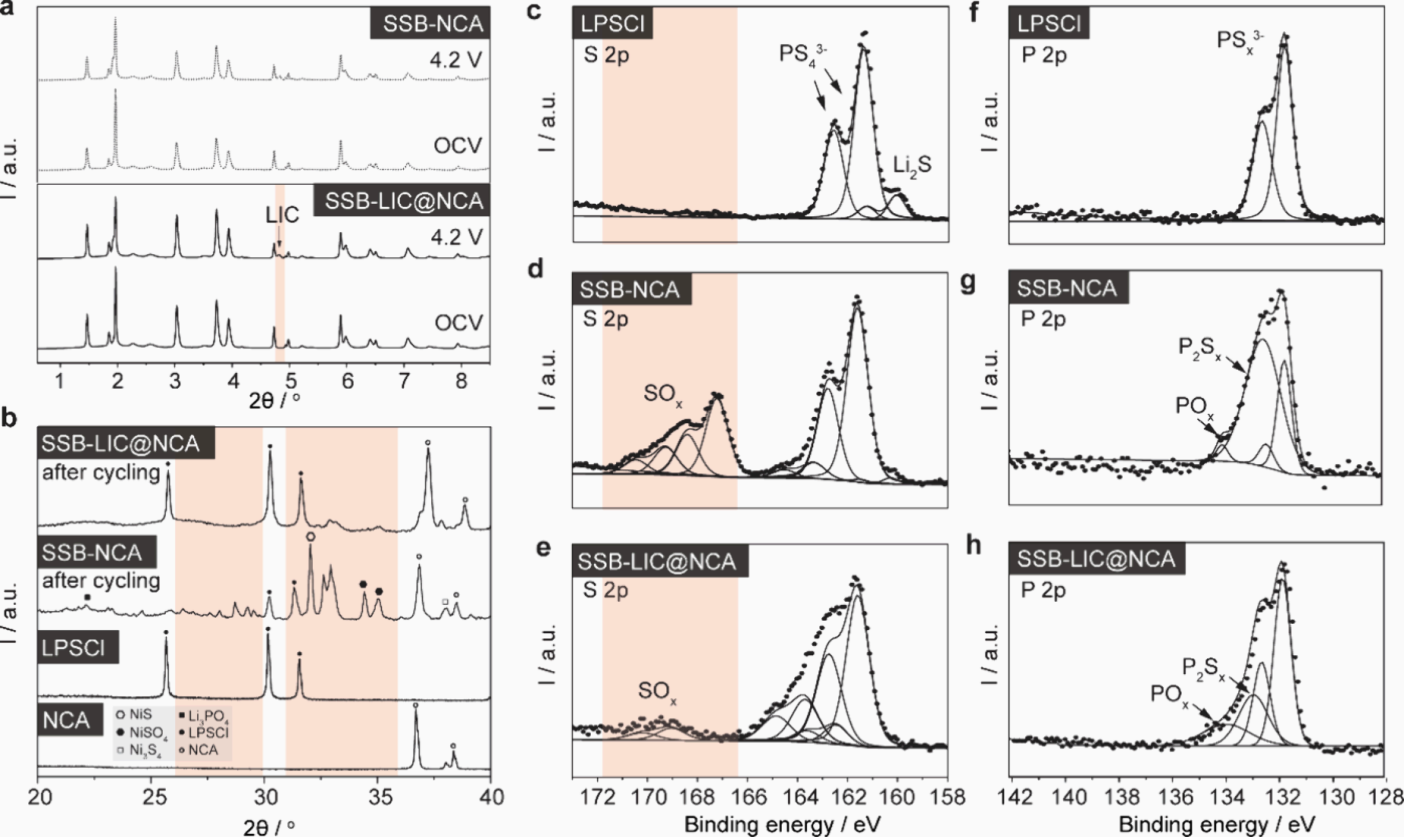
(a) SXRD patterns of cathode composites under various voltages including open circuit voltage (OCV) and 4.2 V. (b) XRD patterns of NCA, LPSCl and cycled cathode composites. Secondary peaks appearing in the XRD spectrum of cycled SSB-NCA (b) are indicative of the decomposition products formed at the NCA|LPSCl interface. XPS spectra of (c–e) S 2p and (f–h) P 2p of LPSCl, cycled NCA composites, and cycled LIC@NCA composites. Cycled SSB-NCA (d, g) exhibits peaks at binding energies beyond 166.0 eV in the S 2p spectrum, signifying the formation of SO_*x*_ compounds, and at 163.5 eV, corresponding to bridging sulfur (P-[S]_*x*_-P). The P 2p spectrum presents new components at 133.1 and 134.5 eV, which are related to the formation of P_2_S_*x*_ (polysulfides) and PO_*x*_. LIC coating (e) suppresses the formation of SO_*x*_ compounds significantly beyond 166 eV while increasing secondary components toward lower binding energies.

Although no phases (Figure 6a) other than NCA and LPSCl have been observed by XRD (potentially related to the detection limit), the degradation of LPSCl and the formation of passivating interphases are evident from XPS and SEM analysis. As shown in Figure 5a,d,e, cathode particles in SSB-NCA are compactly embedded in the LPSCl matrix. The situation changed after 200 cycles when NCA secondary particles were fractured and dispersed into the LPSCl matrix with significant contact loss (Figure 5f–j). Moreover, LPSCl particles alter their shape from a roundish to a needle structure (Figure 5a,b and Figure S1a,b) and significant changes in chemistry take place (Figure 6d,e). For LPSCl powder, the S 2p spectrum shows two doublets. The main peak at 161.4 (S 2p_3/2_) is related to the PS_4_^3–^. A minor second doublet at 160.1 eV (S 2p_3/2_) corresponds to S^2–^, which originates from the Li_2_S precursor.^8^ In the corresponding P 2p spectrum, a single doublet at 131.7 eV (P 2p_3/2_) appears, which can be assigned to the PS_4_^3–^ compounds.^32^ In cycled SSB-NCA, a significant number of new peaks appear at binding energies beyond 166.0 eV in the S 2p spectrum, suggesting the formation of oxygenated sulfur (SO_*x*_) compound,^8^ and at 163.5 eV, related to bridging sulfur (P-[S]_*x*_-P).^33^ The P 2p spectrum presents new components at 133.1 and 134.5 eV, related to the formation of P_2_S_*x*_ (polysulfides) and PO_*x*_,^34^ respectively. Additional peaks indicate the presence of other potentially more complex, compounds. The formation of interphases also contributes to the physical separation of the individual components due to morphological changes (Figure 5f–j).^34^ This heterogeneity potentially increases the nonuniform current distribution around the NCA particles, causing current hotspots. Due to kinetic limitations in the NCA, these hot spots cause local overcharging that leads to high-stress states and ultimately causing fracturing of the cathode particles and void formation (Figure 5i,j and Figure S16).^35^ This fracturing into partially electronic disconnected primary particles and voids results in a lower accessible capacity and subsequently in a higher effective current experienced by NCA particles, which leads, as a consequence, to a further lowering in accessible capacity.^34,35^

For SSB-LIC@NCA, no obvious morphological change, neither for LPSCl (Figure 5c) nor for NCA, has been observed after cycling (Figure 5n) and the physical contact between the individual components remained intact (Figure 5o). EDS mapping reveals an In ring around NCA (Figure 5k–m) after 200 cycles. Additionally, no extra peaks in the In 3d and Cl 2p XPS spectra have been observed pointing toward the stability of LIC (Figure S17). Based on the S 2p and P 2p spectra (Figure 6c–h), it is evident that the LIC coating effectively suppresses the formation of SO_*x*_, and P_2_S_*x*_ and PS_4_^3–^ compounds^36^ (see Supporting Information Note 2 for more details). The improved stability of LIC@NCA compared with NCA is even more prominent in the XRD patterns shown in Figure 6b. The XRD pattern indicates, beside LPSCl and NCA, reflections that can be assigned to Ni_3_S_4_, Li_3_PO_4_, etc. (Figure S18). The characteristic reflections belonging to LIC (Figure S19) do not change with increasing voltage from ∼2 (OCV) to 4.2 V (vs Li/Li^+^), further indicating that LIC is stable and responsible for the improved stability.

## 3 Conclusions

In this study, nanocrystalline halide-based LIC has been tested as a coating for high-voltage cathodes in solid-state batteries to mitigate the degradation of LPSCl, the associated initial discharge capacity loss, poor cycle life, and rate capability.

First, we synthesized nanocrystalline LIC by high-energy ball milling with an average grain size of nanoscale level and a total conductivity of 1.44 mS/cm after a subsequent annealing step at 300 °C. Thereafter, the LIC was coated around NCA particles by a scalable and economically efficient coating process using mechanofusion. Based on mechanofusion, not only a surface smoothing of NCA particles but also a homogeneous and thin coating of about 180 nm have been achieved. Finally, solid-state batteries have been assembled with NCA w/wo LIC coating and tested with respect to their cyclability under different C-rates with a cutoff voltage of 4.2 V (vs Li/Li^+^). It is shown that the LIC coating increases the performance by 80 mAh/g at all C-rates. For example, at 0.1C, the discharge capacity reaches a value as high as 148.3 mAh/g compared to 71.8 mAh/g when no coating has been established. Long-cycling tests deliver stable cycling with 80.1% capacity retention after 200 cycles under 0.2C compared to SSB-NCA with around 40 mAh/g. The improved performance has been identified to be related to the suppression of interfacial side reactions between LPSCl and NCA, interfacial delamination, and particle cracking, as observed for uncoated NCA-based SSBs.

This study demonstrates that (i) LIC is a promising stable solid electrolyte for coating high voltage cathodes to enable sulfide electrolyte-based SSBs when appropriate conditions have been chosen, as well as (ii) that mechanofusion is a viable, easy, and scalable technique for coating spherical-shaped cathode materials by complex compounds, which can play a pivotal role in the material development and their scaling, to enable widespread adoption of future solid-state batteries.

## 4 Experimental Section

### 4.1 Synthesis

LPSCl and NCA were purchased from NEI and used as received. Li_3_InCl_6_ was prepared via a two-step synthesis process: (i) lithium chloride (LiCl, Sigma-Aldrich, 99.9%) and indium chloride (InCl_3_, Sigma-Aldrich, 99.99%) were weighed to the stoichiometric molar ratio (3:1) and mechanically mixed in a ZrO_2_ container with ZrO_2_ balls (diameter = 5 and 10 mm, 1:1, wt %) in a planetary ball mill (Fritsch, Pulverisette 7) at 500 rpm for 24 h, and (ii) the obtained Li_3_InCl_6_ powder (bm-LIC) was pelletized at 300 MPa and further annealed at 300 °C with a heating rate of 2 °C/min for 5 h in Ar atmosphere to obtain final power (an-LIC). The an-LIC was further ball milled for 50 h to prepare a fine LIC powder for mechanofusion.

### 4.2 Coating

A 1 g portion of annealed Li_3_InCl_6_ (LIC) and 19 g of pristine NCA were mixed in mortar and pestle for 30 min to obtain a homogeneous mixture. Thereafter, the powder mixture was further mixed and homogenized in a mechanofusion device (Hosokawa, NOB mode) integrated in a N_2_-filled glovebox (H_2_O and O_2_ < 0.1 ppm) for 2 h under 3600 rpm to achieve LIC-coated NCA particles.

### 4.3 Characterization

The morphologies of LPSCl, Li_3_InCl_6_, and Li_3_InCl_6_-coated NCA (LIC@NCA) were characterized by an FEI Apreo emission scanning electron microscope (SEM) equipped with energy dispersive spectroscopy (EDS). X-ray diffraction (XRD) was performed on a Bruker DAVINCI with a Cu Kα radiation (λ = 1.54178 Å) at room temperature with an airtight holder to avoid air exposure. The data was collected by scanning 1 s per step with a step width of 0.02 from 10 to 70*°* (2θ). The data for the X-ray refinement was collected by scanning 5 s per step with one step of 0.02° from 10 to 70° (2θ). The XRD refinement was performed by using the TOPAS software.

Chemical characterization of the samples and the state of the surface was obtained with X-ray photoemission spectroscopy (XPS). Core level spectra were measured using a monochromatized Al Kα (1486.6 eV) line with a hemispherical analyzer at NTNU XPS facilities and a Mg Kα (1253.6 eV) nonmonochromatized light and a Specs Phoibos 150 hemispherical analyzer at UAM. Measurements were performed at room temperature and with chamber pressures below 10^–9^ mbar. The calibration of the binding energy was performed using C 1s and Au 4f reference peaks. A Shirley background and asymmetric singlet pseudo-Voigt function was used to fit the line shape of core levels. The fit was optimized using a Levenberg–Marquardt algorithm^37^ and a normalized χ^2^ reliability factor.

### 4.4 Electrochemical Measurements

The Li-ion conductivity was measured by adding 0.15 g of LIC or LPSCl (NEI corporation) powder into a PEEK (polyether ether ketone) cell with a diameter of 10 mm and further pressed at 300 MPa. EIS measurements were carried out at 375 MPa and room temperature (25 °C, unless specified) in the frequency range of 10 to 7 MHz with 10 mV of applied sinus amplitude. For half cells, first, 80 mg of LPSCl was added in the PEEK cell with a diameter of 10 mm and pressed at 375 MPa. The cathode composite was prepared by hand-mixing the NCA (LIC@NCA) and LPSCl in a ratio of 7:3 (wt %) in an agate mortar and pestle inside an Ar-filled glovebox. Nine to thirteen mg of as-prepared cathode composite powder was placed on one side of the LPSCl pellet and pressed at 375 MPa.

To prepare the Li–In alloy anode (Li_0.5_In), CR2016 coin cells were assembled with In as the cathode, Li as the anode, and a liquid electrolyte (1 M LiTFSI in DOL and DME in a 1:1 vol. ratio). After 40 h of Li plating at a current density of 0.25 mA/cm^2^ (Figure S8), the Li–In disk was obtained after cell disassembling, washing with DME, and subsequent drying at 50 °C in an oven inside Ar-filled glovebox. Lastly, the Li–In disk (10 mm diameter) was attached to another side of the LPSCl pellet and pressed at 150 MPa. All the half-cell SSBs were assembled inside an Ar-filled glovebox (H_2_O < 0.1 ppm, O_2_ < 0.1 ppm). The half cells were tested at room temperature (25 °C) and 120 MPa pressure for EIS and GCPL at different C rates (1C = 160 mAh/g) using VMP-300 potentiostat (BioLogic) and Neware battery cycler.

### 4.5 Operando XRD

For operando XRD measurements, the in situ cell with a 3 mm diameter was assembled with 1.2 mg of cathode composite, 7 mg of LPSCl, and Li–In disk. The XRD patterns were collected in a transition mode at beamline SNBL-BM31 at the European Synchrotron Radiation Facility (ESRF) (Swiss-Norwegian Beamline, European Synchrotron Radiation Facility, Grenoble, France) using a monochromatic high brilliant X-ray beam with λ = 0.244860 Å (data are available in ref (38)). The diffractometer is based on a Pilatus2M detector^39^ and data processing was done using the BUBBLES software. Before operando measurements, the beam position was optimized to hit more of the cathode side than of the electrolyte.

Data evaluation was performed using TOPAS V6 software. The PEEK polymer of the cell was modeled with a single peak phase consisting of 12 single reflections, peak positions, and shapes were extracted from measurements on the empty cell and fixed during multidata set processing, allowing only the integrated intensity to vary. Peak shapes of the main phases LPSC and NMC were modeled by the Rietveld method using the Thompson–Cox–Hastings pseudo-Voigt function.

## Supporting Information Available

SEM images of LPSCl, NCA, LIC@NCA, and cathode composites; (S)XRD and corresponding refinements of LPSCl, LIC, NCA, LIC@NCA, PEEK, and cycled composites; lattice parameters; XPS of pristine and cycled cathode composites; particle size distributions of NCA and LIC@NCA (PDF)
