# Supplementary material for: Elucidating the Impact of Li3InCl6-Coated LiNi0.8Co0.15Al0.05O2 on the Electro-Chemo-Mechanics of Li6PS5Cl-Based Solid-State Batteries
Source: Chem Mater. 2024 Jun 13;36(12):6017–26. doi: 10.1021/acs.chemmater.4c00515 (PMC11209938; doi:10.1021/acs.chemmater.4c00515)
Supplement: Supplementary file 1 — cm4c00515_si_001.pdf [file cm4c00515_si_001.pdf]

Supplementary information

Elucidating the Impact of Li_3InCl_6 -Coated $LiNi_{0.8}Co_{0.15}Al_{0.05}O_2$ on the Electro-Chemo-Mechanics of Li_6PS_5Cl-Based Solid-State Batteries

Feng Jin[1], Laras Fadillah[1], Hung Quoc Nguyen[1], Torgeir Matre Sandvik[1], Yu Liu[1], Adrián García-Martín[2,3], Elena Salagre[2,3], Enrique G. Michel[2,3], Dragos Stoian[4], Kenneth Marshall[4], Wouter Van Beek[4], Guenther Redhammer[5], Mir Mehraj Ud Din[1,6], and Daniel Rettenwander[1,6**]*

[1]Department of Material Science and Engineering, NTNU Norwegian University of Science and Technology, Trondheim, Norway
[2]Departamento de Fisica de la Materia Condensada, Facultad de Ciencias, Universidad Autónoma de Madrid, Madrid, Spain
[3]Condensed Matter Physics Center (IFIMAC), Universidad Autónoma de Madrid, Madrid, Spain
[4]Swiss-Norwegian Beam Lines at European Synchrotron Radiation Facility, 71 Avenue des Martyrs, 38000 Grenoble, France
[5]Department of Chemistry and Physics of Materials, University of Salzburg, 5020, Salzburg, Austria
[6]Christian Doppler Laboratory for Solid-State Batteries, NTNU Norwegian University of Science and Technology, Trondheim, Norway

[*]Corresponding Author: mir.m.u.din@ntnu.no

[**]Corresponding Author: daniel.rettenwander@ntnu.no

Supplementary Note 1: Operando X-ray diffraction

Evaluation of ESRF data were performed using the TOPAS V.6 software by Rietveld method. The background of the diffraction pattern was modeled with a Chebychev polynomial of 3rd order, and the Peak shapes of the main crystalline phases Li_6PS_5Cl (LPSCl), $LiNi_{0.8}Co_{0.15}Al_{0.05}O_2$ (NCA), and Li_3InCl_6 (LIC) were refined using the Thompson-Cox-Hastings pseudo-Voigt function (TCH-PV) for the peak shapes. No crystallite size or strain parameters were used at this stage. Structural data for LPSCl were taken from[1] (ICSD code 133976), for NCA from (ICSD code 257247), and for LIC from (ICSD code 17638[2]).

The PEEK polymer of the cell also diffracts, and its pattern was modeled with a peak phase consisting of 12 single reflections. Peak positions, shapes (TCH-PV), and intensity of it were extracted from measurements on the empty cell (see Figure S14). Peak positions and line width parameters of PEEK were then fixed during multi-dataset processing of the working cell, allowing only the integrated intensity to vary for the PEEK phase.

Structural parameters for LPSCl, NCA, and LIC were refined on the ready-prepared cell before charging (pristine cell), including atomic positions and isotropic atomic displacement parameters. These data were taken as a starting point for the subsequent sequential refinements. During these refinements, the line width parameters, atomic positions, and atomic displacement parameters of the LPSCl, NCA, and LIC were fixed to the ones determined for the pristine cell and only scale factors and lattice parameters were allowed to vary freely. This is done to reduce the amount of refinement parameters and to get stable refinements over the whole set of data. In free refinements, however, no systematic changes in these structural parameters were found, but the convergence was slow.

To account for any line broadening during electrochemical treatment, crystallite size, and stain parameters were allowed to refine whenever the line-width parameters were fixed to the ones obtained for the pristine cell, but there was neither some significant size broadening nor an observed micro-strain. During the whole cycling process, no changes in lattice parameters and relative weight fractions are observed for LPSCl, indicating that there is no alteration of the electrolyte macroscopically during electrochemical testing.

The reaction of NCA during cycling is to be described as a two-phase reaction.[3] Phase R1 is the pristine NCA and some fraction remains over the whole cycling period(s) without significant changes in unit cell parameters, its amount is slightly decreasing. At some early point of electrochemical treatment, a second NCA phase (R2) must be introduced. R2 shows a

solid-solution behavior with distinct shifts in peak positions and thus changes in lattice parameters as discussed in the main text and below.

It is shown that peaks of the R2 phase, belonging to (003) and (101) change during charge and discharge in the initial cycle. Specifically, the peak for (003) shifted to a low angle with Li^+ extraction from Li-layers and further resulted in increasing electrostatic repulsion between adjacent oxygen atoms during the charging process. The shifting of (101) peak towards higher angles is associated with a continuous shrinkage of the *a*-axis and *b*-axis due to the reduced ionic radii in TM layers, and vice versa.

Supplementary Note 2: XPS

We describe here in more detail the new S 2p and P 2p components that appear after cycling the solid-state batteries employing NCA with (SSB-LIC@NCA) and without LIC coating (SSB-LIC@NCA) (Fig. S11 and S12).

The S 2p spectra from LPSCl presents a single component attributed to PS_4^{3-}, i.e., S atoms in the electrolyte. This is the major S component in all samples. A minor component appears near 160 eV for LPSCl and SSB-NCA, which is attributed to S^{2-} originating from the Li_2S precursor.

The S 2p spectrum for cycled SSB-NCA presents two new components at higher binding energies (167.2 eV and 169.3 eV) that are related to the formation of oxygenated sulfur (SO_x) compounds. The component at 167.2 eV may be present also in the LPSCl spectrum, but in a much smaller proportion. An additional minor component related probably to bridging S (P-$[S]_x$-P) is observed at 163.3 eV. An inspection of the spectrum corresponding to cycled SSB-LIC@NCA reveals that the component at 167.2 eV disappears, while the one at 169.3 eV strongly decreases, indicating that the LIC coating is suppressing their formation. At the same time, the presence of the LIC seems to cause the formation of other extra phases compared to NCA without coating. One extra phase (around 163.5 eV) is one which was already present in cycled SSB-NCA, while the second phase (near 162.5 eV) is difficult to identify, since it lies at the edge of the peak corresponding to the major component. We believe that these minor secondary phases form a stable SEI that improves the cycling performance of the SSB-LIC@NCA cell.

The P 2p spectrum from LPSCl contains a single component corresponding to PS_4^{3-} and appearing near 131.7 eV, which is the major component in the spectra of the cycled samples as well. The spectrum of cycled SBB-NCA presents two new minor components at higher

binding energies (at 133.1 eV and 134.5 eV) attributed to the formation of P_2S_x (polysulfides) and PO_x, respectively. The P 2p spectrum corresponding to cycled SSB-LIC@NCA is characterized by a larger component at 134.5 eV, and two close components at 133.1 eV and 132.5 eV, the second one superimposed with the major component at 131.7 eV. Interestingly, a new component corresponding to more reduced P is observed at 130.9 eV. These changes are consistent with the formation of a protective layer that contains reaction products that do not interfere with the battery performance.

To further evaluate the oxidation stability of Li_3InCl_6, The In 3d spectrum of SSB-LIC@NCA operated with 4.5 V shown in the Fig. S11. There are two shoulder peaks emerged at eV and eV, which are assigned to In_2O_3 [4]. On the contrast, there is no corresponding peaks observed in In 3d spectrum of SSB-LIC@NCA operated with 4.4 V.

FIGURES

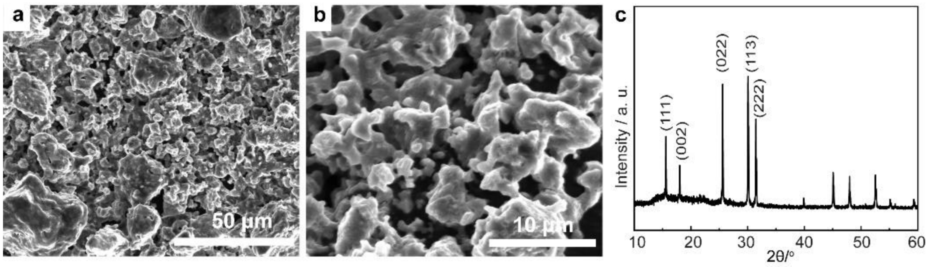

Figure S1. (a,b) SEM images of LPSCl; (c) XRD pattern of LPSCl.

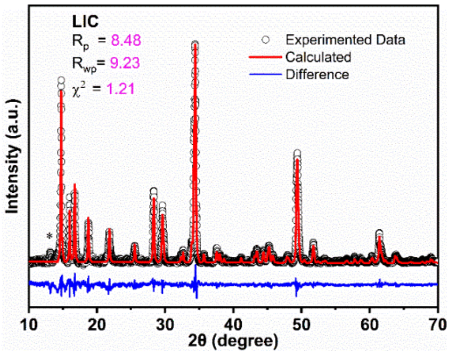

Figure S2. High-resolution XRD pattern and Rietveld refinement result of Li_3InCl_6 (LIC). The XRD patterns of LIC could be indexed well with the distorted rock-salt structure in monoclinic space group C2/m (ICSD No. 04-009-9027). a= 6.40800 Å, b=11.07910 Å, c=6.38200 Å, α=γ= 90.0000°, β=109.8610°.

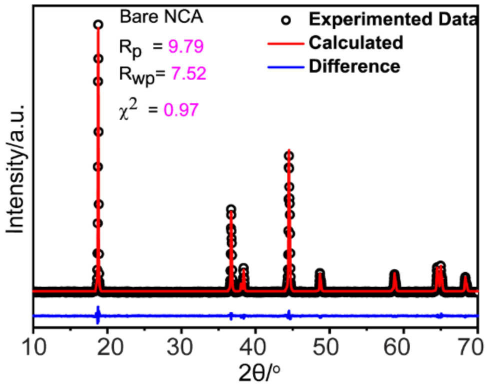

Figure S3. High-resolution XRD pattern collected from the bare NCA particles and corresponding Rietveld refinement plot.

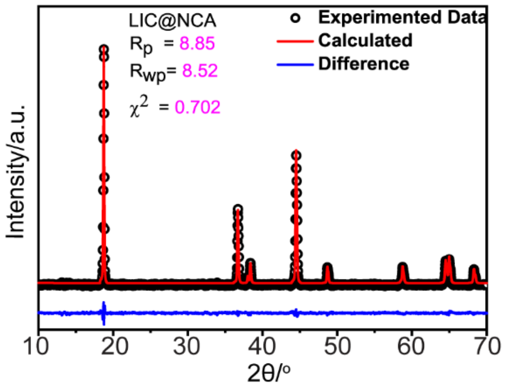

Figure S4. High-resolution XRD pattern collected from the LIC@NCA particles and corresponding Rietveld refinement plot.

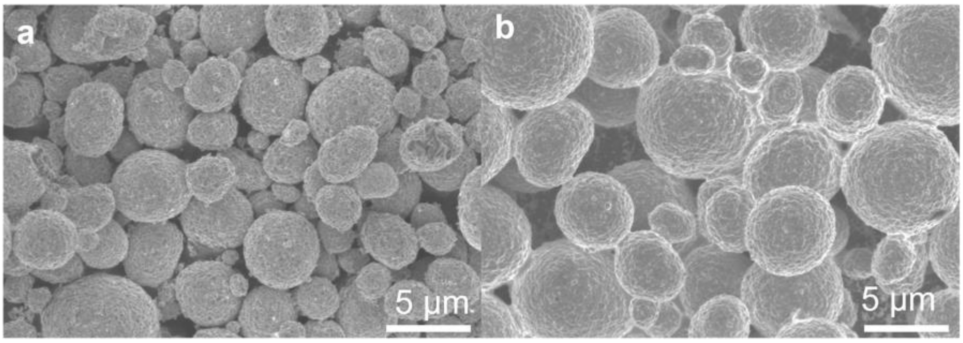

Figure S5. Low-magnification SEM images of the bare NCA (a) and LIC@NCA (b) particles.

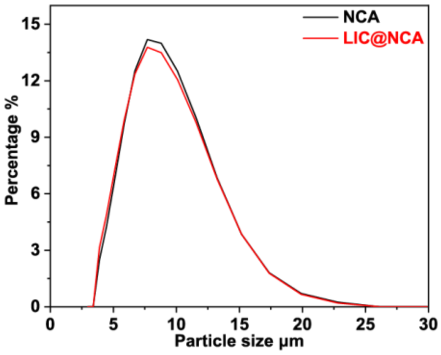

Figure S6. Particle size distribution of pristine NCA and LIC@NCA.

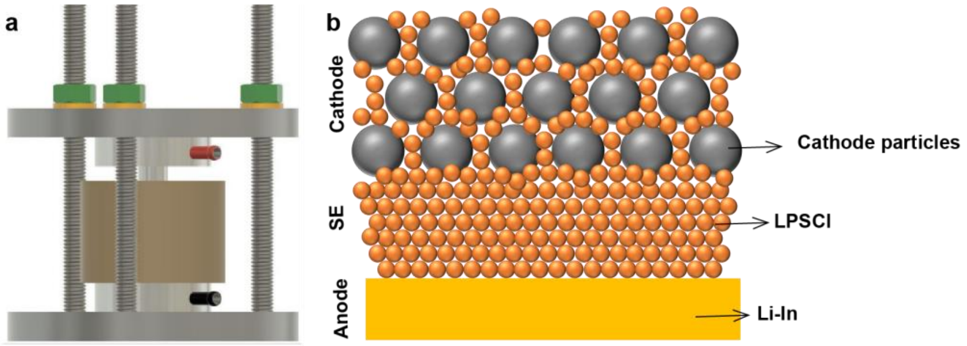

Figure S7. (a) Set up for SSBs test illustrating the design of peek cell SSB, consisting of an outer peek insulation casing and two stainless-steel plungers for positive and negative electrode connections, integrated in a stainless-steel pressure holder framework. (b) Schematic illustration of a typical SSB comprising of Li-In anode, compacted LPSCl solid electrolyte and a composite cathode.

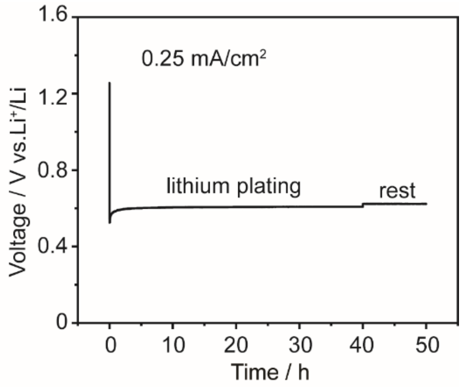

Figure S8. Lithium plating curve on indium disk.

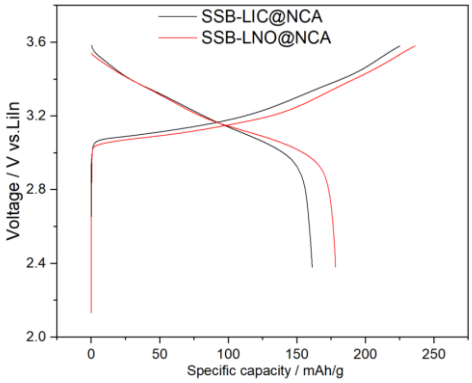

Figure S9. Charge and discharge curves of SSB-LIC@NCA and SSB-LNO@NCA.

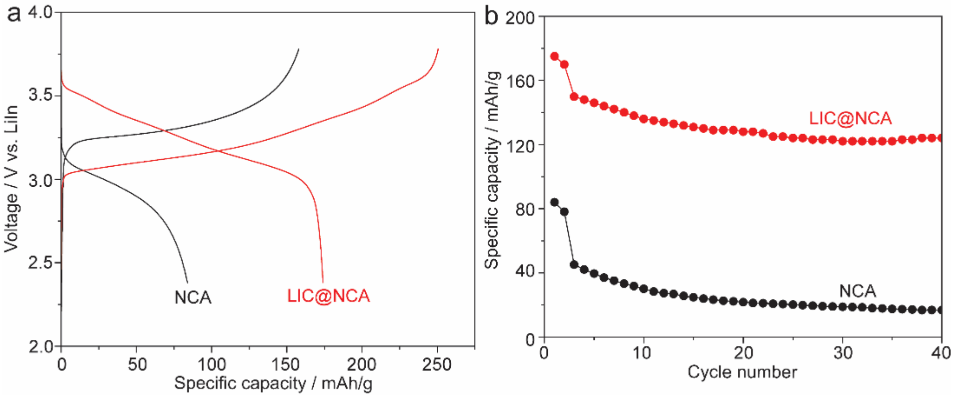

Figure S10. SSB-NCA and SSB-LIC@NCA cells performance with cut-off voltage of 4.4 V vs Li/Li$^+$. (a) Voltage profiles of the pristine NCA and LIC@NCA for the first cycle at 0.1C (b) Corresponding long-term cycling behavior at 0.2 C.

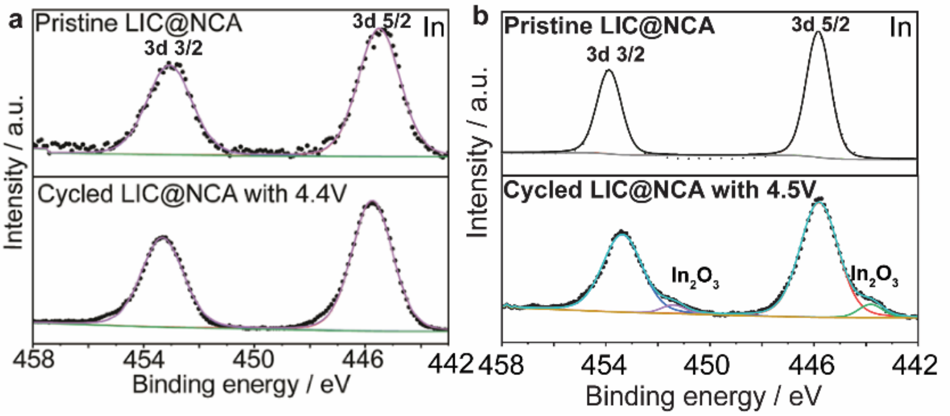

Figure S11. XPS spectra of In 3d of cycled LIC@NCA composites with cut-off voltage of 4.4 V(a) and 4.5 V(b) vs Li/Li$^+$.

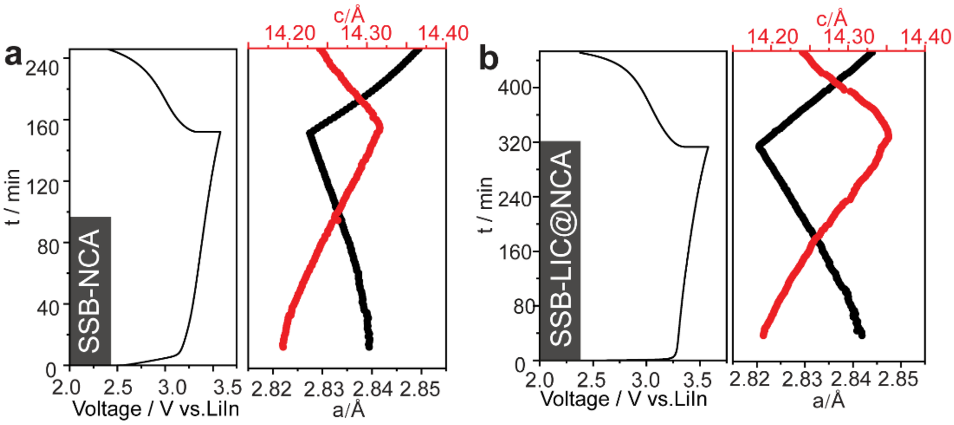

Figure S12. Lattice parameter changes including a and c during first charge-discharge cycle for SSB-NCA and SSB-LIC@NCA cell of SXRD test.

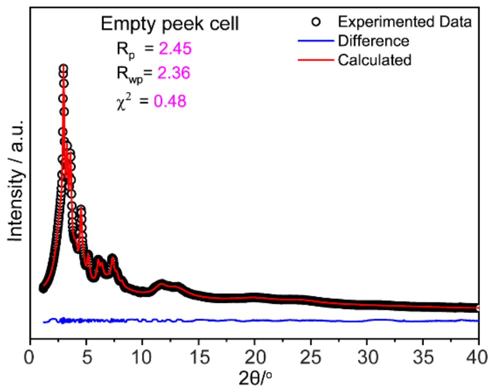

Figure S13. Diffraction data of the empty cell showing the PEEK polymer, fitted with single peaks without structural information and a polynomial function of 3rd order for the background. Data collected at BM31@ESRF with λ = 0.244860 Å.

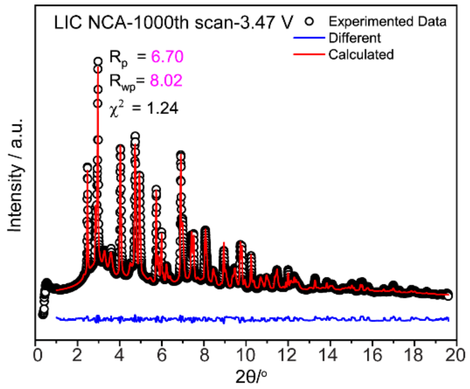

Figure S14. Typical synchrotron PDX pattern of a cell prepared with LIC@NCA as the active material, data collected at BM31@ESRF λ = 0.244860 Å for the 1st charging cycle.

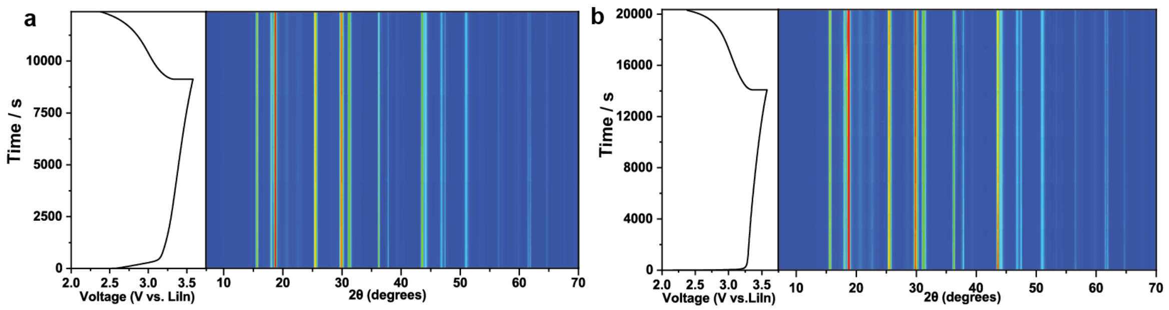

Figure S15. Contour plot showing the evolution of Bragg reflections and corresponding voltage profile as a function of x(Li) for SSB-NCA (a) and SSB-LIC@NCA (b).

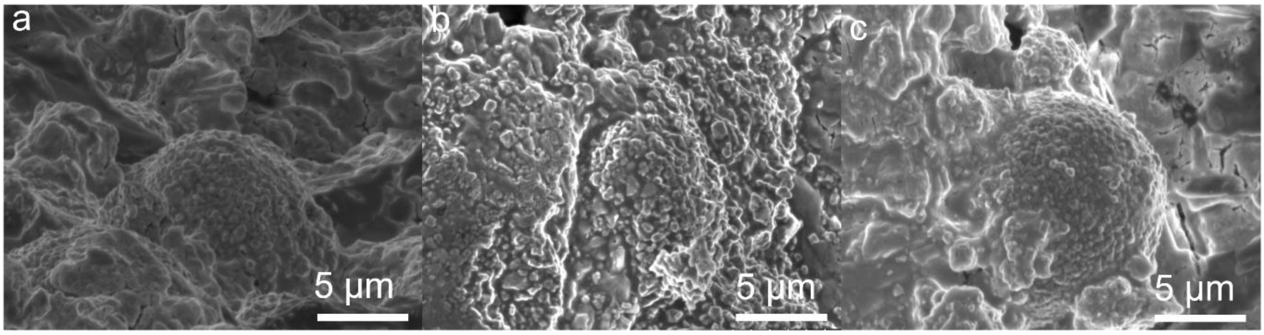

Figure S16. Cross-section view (a-c) SEM images of cathodes with bare NCA and LIC@NCA before and after cycling (200 cycles).

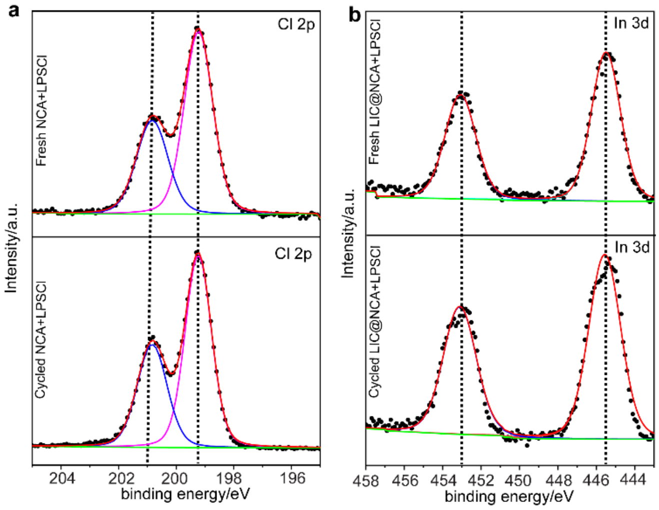

Figure S17. XPS spectra of (a) Cl 2p and (b) In 3d of fresh LIC@NCA composites and cycled LIC@NCA composites.

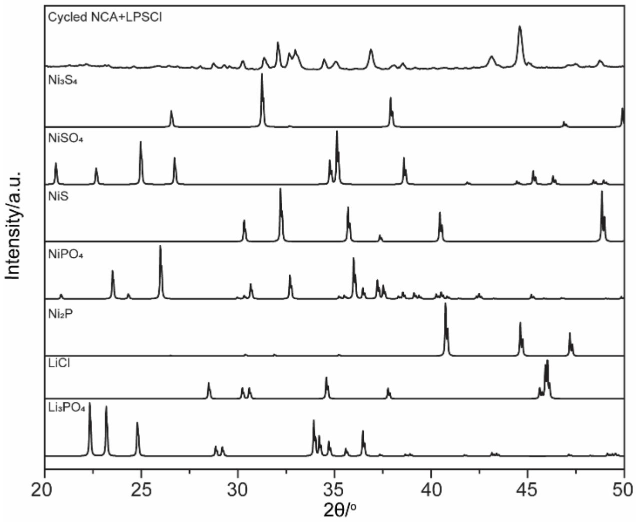

Figure S18. XRD patterns of NCA after cycling compared to some reference compounds.

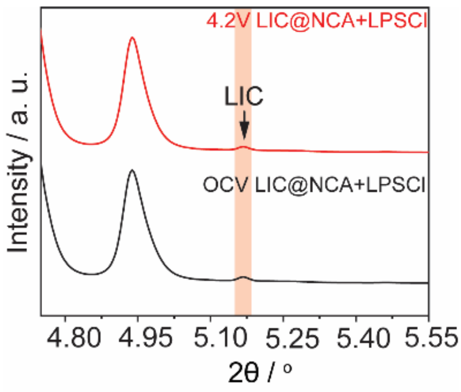

Figure S19. SXRD patterns of LIC@NCA cathode composites under different voltage.

TABLES

Table S1 Rietveld analysis of the XRD pattern of bare NCA.

		X	Y	Z	Occ	U	Site	Sym
Li	Li1	0.00000	0.00000	0.50000	1.000	0.011	3b	-3m
Al	Al1	0.00000	0.00000	0.00000	0.148	0.021	3a	-3m
O	O1	0.00000	0.00000	0.25910	1.000	0.008	6c	3m
Ni	Ni2	0.00000	0.00000	0.00000	0.700	0.012	3a	-3m
Co	Co1	0.00000	0.00000	0.00000	0.150	0.000	3a	-3m
Ni	Ni1	0.00000	0.00000	0.50000	0.010	0.000	3b	-3m

Table S2. Rietveld analysis of the XRD pattern of bare NCA.

		X	Y	Z	Occ	U	Site	Sym
Li	Li1	0.00000	0.00000	0.50000	1.000	0.011	3b	-3m
Al	Al1	0.00000	0.00000	0.00000	0.148	0.021	3a	-3m
O	O1	0.00000	0.00000	0.25910	1.000	0.008	6c	3m
Ni	Ni2	0.00000	0.00000	0.00000	0.700	0.012	3a	-3m
Co	Co1	0.00000	0.00000	0.00000	0.150	0.000	3a	-3m
Ni	Ni1	0.00000	0.00000	0.50000	0.010	0.000	3b	-3m

Table S3. Rietveld analysis of the XRD pattern of LIC@NCA.

		X	Y	Z	Occ	U	Site	Sym
Li	Li1	0.00000	0.00000	0.50000	0.990	0.000	3b	-3m
Al	Al1	0.00000	0.00000	0.00000	0.150	0.000	3a	-3m
O	O1	0.00000	0.00000	0.25910	1.000	0.000	6c	3m
Ni	Ni2	0.00000	0.00000	0.00000	0.700	0.000	3a	-3m
Co	Co1	0.00000	0.00000	0.00000	0.150	0.000	3a	-3m
Ni	Ni1	0.00000	0.00000	0.50000	0.010	0.000	3b	-3m

Table S4. Refined structural parameters for the bare NCA and LIC@NCA CAM particles.

samples	a/Å	c/Å	V/Å3
Bare NCA	2.86750	14.18810	101.032649
LIC@NCA	2.86697	14.18430	100.968259

References

[1] Schlenker, R.; Hansen, A. L.; Senyshyn, A.; Zinkevich, T.; Knapp, M.; Hupfer, T.; Ehrenberg, H.; Indris, S.; *Chem.Mater.* **2020**, *32*, 8420-8430.

[2] Li, X. N.; Liang, J. W.; Chen, N.; Luo, J.; Adair, K. R.; Wang, C. H.; Banis, M. N.; Sham, T. K.; Zhang, L.; Zhao, S. Q.; Lu, S. G.; Huang, H.; Li, R. Y.; Sun, X. L. *Angew.Chem.Int.Ed.* **2019**, *58*, 16427-16432.

[3] a)Grenier,A.; Liu, H.; Wiaderek, K. M.; Lebens-Higgins, Z. W.; Borkiewicz, O. J.; Piper, L. F. J.; Chupas, P. J.; Chapman, K. W.*Chem.Mater.***2017**, *29*, 7345-7352; b) Robert, R.; Bünzli, C.; Berg, E. J.; Novák, P. *Chem.Mater.* **2015**, *27*, 526-536; c) Yoon, W.-S.; Chung, K. Y.; McBreen, J.; Yang, X.-Q. *Electro. Commun.* **2006**, *8*, 1257-1262.

[4] Zhang, A.-B.; Wang, J.; Yu,R.-Z.; Zhuo, H.-X.; Wang, C.-H.; Ren, Z.-M.; Wang, J.-T. Practical Application of Li-Rich Materials in Halide All-Solid-State Batteries and Interfacial Reactions between Cathodes and Electrolytes. *ACS Appl. Mater. Inter.* **2023**, *15*, 8190-8199.
